# Hydrophobic solution functions as a multifaceted mosquito repellent by enhancing chemical transfer, altering object tracking, and forming aversive memory

**DOI:** 10.1038/s41598-024-55975-w

**Published:** 2024-03-05

**Authors:** Bianca M. Wiedemann, Kohei Takeuchi, Kazumi Ohta, Aya Kato-Namba, Masayuki Yabuki, Hokto Kazama, Takao Nakagawa

**Affiliations:** 1 Human Health Care Products Research, Kao Corporation, 2‑1‑3 Bunka, Sumida, Tokyo, 131‑8501 Japan; 2 Sensory Science Research, Kao Corporation, 2‑1‑3 Bunka, Sumida, Tokyo, 131‑8501 Japan; 3 RIKEN Center for Brain Science, 2-1 Hirosawa, Wako, Saitama 351-0198 Japan; 4 RIKEN CBS-KAO Collaboration Center, 2-1 Hirosawa, Wako, Saitama 351-0198 Japan; 5 Graduate School of Arts and Sciences, The University of Tokyo, 3-8-1 Komaba, Meguro-ku, Tokyo, 153-8902 Japan

**Keywords:** Behavioural ecology, Physical chemistry, Malaria, Viral infection

## Abstract

Developing a safe and potent repellent of mosquitoes applicable to human skins is an effective measure against the spread of mosquito-borne diseases. Recently, we have identified that hydrophobic solutions such as low viscosity polydimethylsiloxane (L-PDMS) spread on a human skin prevent mosquitoes from staying on and biting it. This is likely due to the ability of L-PDMS in wetting mosquito legs and exerting a capillary force from which the mosquitoes attempt to escape. Here we show three additional functions of L-PDMS that can contribute to repel *Aedes albopictus*, by combining physicochemical analysis and behavioral assays in both an arm cage and a virtual flight arena. First, L-PDMS, when mixed with topical repellents and applied on a human skin, enhances the effect of topical repellents in reducing mosquito bites by efficiently transferring them to mosquito legs upon contact. Second, L-PDMS applied to mosquito tarsi compromises visual object tracking during flight, exerting an influence outlasting the contact. Finally, L-PDMS applied to mosquito tarsi acts as an aversive reinforcer in associative learning, making mosquitoes avoid the conditioned odor. These results uncover a multifaceted potential of L-PDMS in altering a sequence of mosquito behaviors from biting a human skin, visual object tracking following takeoff, to the response to an odor linked with L-PDMS.

## Introduction

Mosquito-borne diseases are posing an increasing threat to humans worldwide. Despite great efforts to control outbreaks, Malaria and Dengue fever see reoccurring waves of infection^1,2^. As personal protection using mosquito repellents is a major defense against infection, development of more effective repellents represents a potent measure towards decreasing a risk of mosquito-borne infections.

Commonly, volatile materials such as citronella oil (*Cymbopogon winterianus*) and *N,N*-dimethyl-meta-toluamide (DEET) are utilized as topical repellents. These prevent mosquitoes from approaching a host, by interacting with olfactory receptors^3^. The olfactory co-receptor Orco is susceptible for DEET, but mutation prevents this specific interaction^4^. Subsequently, orco mutant mosquitoes are not repellent by volatile DEET^5^. Nonetheless, it can still be sensed by mosquito tarsi, likely via gustatory receptors, ionotropic receptors, and transient receptor potential (TRP) channels expressed in the periphery^5–8^. Although it is unknown how citronella oil is detected by mosquito tarsi, it activates TRP receptors in *Drosophila*^9,10^. In search of novel repellents, various candidates have been identified, which all have high volatility to achieve efficient mosquito repellence, hence relying on detection via olfactory receptors^11–16^.

Recently, we identified that hydrophobic liquids induce an escape response in mosquitoes after tarsal contact^17^. Low viscosity polydimethylsiloxane (L-PDMS) forms a low contact angle on hydrophobic surfaces including the scales of mosquitoes and efficiently wets mosquito legs upon contact. This wetting generates a capillary force, pulling mosquito’s legs towards the liquid. As mosquitoes need to escape from this force, when applied on a human skin, L-PDMS prevents the mosquitoes from frequently contacting and biting the skin.

Here, using *Aedes albopictus,* we explored the potential of L-PDMS as a repellent in three additional aspects. First, given that L-PDMS easily wets mosquitoes, we examined whether its mixture with topical repellents like citronella oil and DEET can be used to more effectively apply topical repellents on mosquito legs. If so, the mixture of L-PDMS and topical repellents spread on a human skin should be more effective than the repellents alone in reducing mosquito bites. Second, we examined if L-PDMS affected the flight of mosquito after the tarsal contact, exerting a behavioral effect outlasting the contact. Third, we examined if the aversive experience of contacting the L-PDMS is sufficient to confer a negative meaning to an accompanying odor, such that the odor alone can be used as a longer lasting repellent. We found evidence in support of all of these hypotheses, illustrating the potential of L-PDMS in altering a sequence of mosquito behaviors from landing and biting a human skin, visual object tracking after takeoff, to the response to the olfactory cue associated with L-PDMS.

## Results

### L-PDMS prevents mosquito bites by effectively transferring topical repellents to mosquito legs upon contact

**Figure 1:**
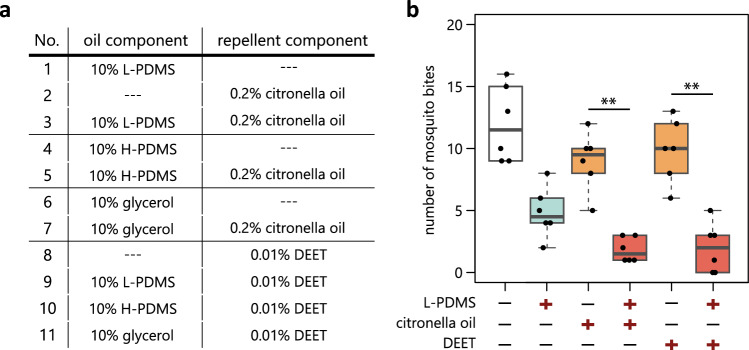
L-PDMS enhances the effect of citronella oil and DEET in reducing mosquito bites. (**a**) Solutions used in the study. Ethanol was used as solvent. (**b**) Number of mosquito bites on a human arm treated with different solutions (n = 6 experiments; one-way ANOVA, p = 1.79E-10; Tukey’s HSD test: citronella oil vs L-PDMS and citronella oil, p = 0.0022, DEET vs L-PDMS and DEET, p = 0.0005).

We started by testing our hypothesis that a mixture of L-PDMS and topical repellents spread on a human skin wets the mosquito legs more effectively than the repellents alone and thus more strongly prevents mosquito bites. We asked human subjects to place one of their arms treated with various mixtures of oils and topical repellents (Fig. 1a) in a cage housing 25 female mosquitoes, and counted the number of mosquito bites after 10 min (Fig. 1b). Three different oils were tested at a concentration of 10% in ethanol: L-PDMS (6cs), high viscosity PDMS (H-PDMS, 5000cs), and glycerol. For repellents, we selected 0.2% citronella oil and 0.01% DEET. As expected, we found that mixtures of L-PDMS and repellents more potently prevented mosquito bites as compared to the repellents alone (Fig. 1b). Importantly, the effects of mixtures were stronger than L-PDMS alone although the magnitude did not reach a significance for the mixture with DEET (Fig. 1b). H-PDMS and glycerol, on the other hand, did not exhibit such facilitating effects (Fig. S1).

**Figure 2:**
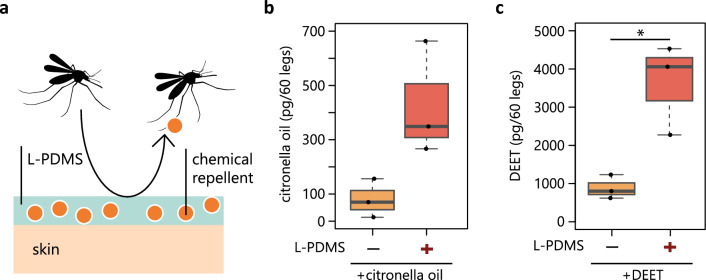
L-PDMS transfers the topical repellents more efficiently to mosquito legs. (**a**) Schematic of a hypothesis where L-PDMS increases mosquito repellence through enhanced, direct transfer of topical repellents from a skin to its legs. (**b**) The amount of citronellal transferred to mosquito legs tend to increase with an addition of L-PDMS (n = 3, t-test, p = 0.07). (**c**) The amount of DEET transferred to mosquito legs increases with an addition of L-PDMS (n = 3, t-test, p = 0.04).

We investigated if more repellents were indeed transferred to mosquito legs when they are mixed with L-PDMS. We made isolated mosquito legs come into contact with an artificial skin treated with different solutions and subsequently measured the amount of repellents on the legs using gas chromatography mass spectrometry (GC–MS) (Fig. 2a–c). Because citronella oil is a complex mixture of different molecules, we focused on one of the components, citronellal, for the analysis. We detected a larger amount of citronellal on legs when they were treated with a mixture of L-PDMS and citronella oil as compared to pure citronella oil (Fig. 2b). This value was much larger than the case of treatment with mixtures of H-PDMS or glycerol (Fig. S2). Similar results were obtained with DEET (Fig. 2c).

These results suggest that L-PDMS prevents mosquito bites by effectively transferring topical repellents to mosquito legs upon contact. 

### L-PDMS decreases the accuracy of visual object tracking during flight

Following a contact with L-PDMS, mosquitoes exhibit grooming behavior as if they are trying to wipe off the solution on their legs (supplemental movie 1), suggesting that L-PDMS exerts prolonged aversive effects on mosquitoes. Therefore, we asked whether L-PDMS continues to compromise some aspects of mosquito behavior.

**Figure 3:**
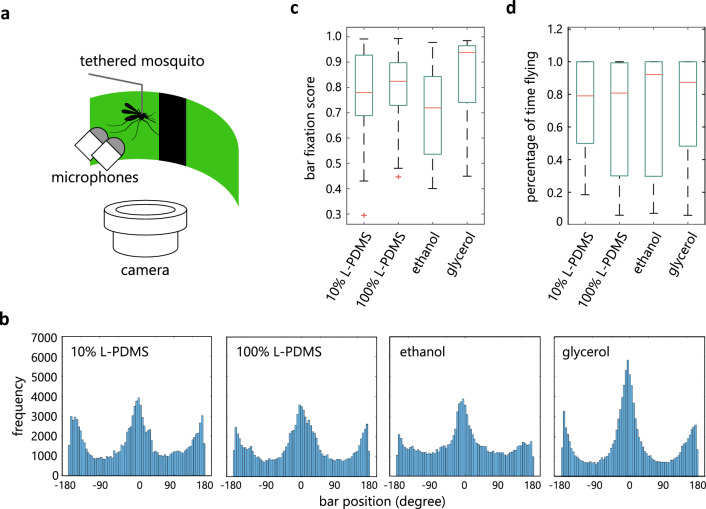
L-PDMS decreases the accuracy of visual object tracking during flight. (**a**) Schematic of a flight arena. (**b**) Histograms of bar position, which reflect the accuracy of bar tracking by flying mosquitoes whose tarsi are treated with different solutions. Each histogram represents an average of multiple mosquitoes (n = 25, 17, 22, and 15 mosquitoes for 10% L-PDMS, 100% L-PDMS, 100% ethanol, and 100% glycerol). (**c**) A bar fixation score quantifying the accuracy of bar tracking (see “Methods” for the definition). The scores are significantly different between solutions (Kurskal–Wallis test, p = 0.03) with ethanol being smaller than glycerol (Dunn–Sidak test, p = 0.02). (**d**) The percentage of time flying is similar across solutions (Kurskal–Wallis test, p = 0.42).

Specifically, we examined the ability of mosquitoes to track a visual object during flight, which is critical in pursuing and landing on hosts. Mosquito’s flight was monitored with high spatiotemporal resolution in the virtual flight arena previously developed to characterize multisensory cue-based maneuver of *Drosophila melanogaster* (Fig. 3a^18,19^). Individual mosquitoes were placed in the arena that provides visual stimuli with an array of LEDs. Visual stimuli were updated depending on the turning behavior assessed by the difference between the sound levels of left and right wingbeats recorded by a pair of microphones. This differential sound level is correlated with the difference between wingbeat amplitudes, a proxy for yaw torque^20^.

We presented a dark, vertical bar in closed-loop and examined how well mosquitoes tracked it after 10% or 100% L-PDMS was applied to their tarsi (Fig. 3b,c). We also examined the behavior after applying 100% ethanol, another solution that efficiently wets mosquito legs, and 100% glycerol that does not. We found that although mosquitoes treated with different solutions flew for similar periods of time (Fig. 3d), they tracked the bar with different accuracy (Fig. 3b,c). Specifically, mosquitoes treated with ethanol tracked the bar less accurately as compared to those treated with glycerol (Fig. 3b,c). Similar tendency was observed with L-PDMS treatment (Fig. 3b,c), suggesting that solutions that efficiently wet the hydrophobic mosquito legs decrease the mosquito’s ability to track a visual object. 

### L-PDMS drives aversive olfactory learning

**Figure 4:**
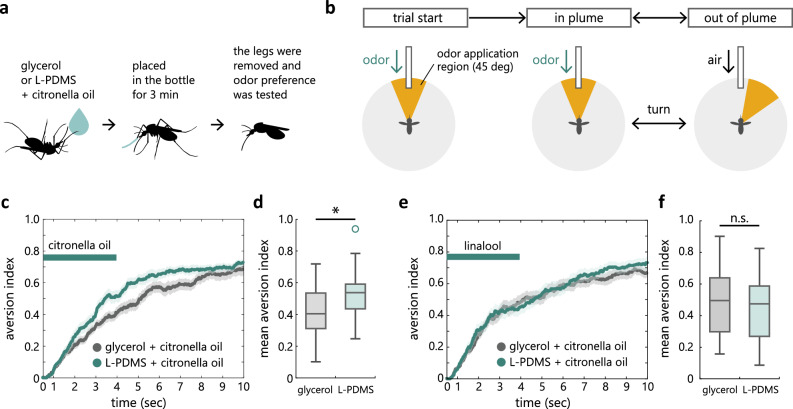
L-PDMS drives aversive olfactory learning. (**a**) Schematic of an experimental procedure. A mixture of 100% L-PDMS and 0.2% citronella oil or 100% glycerol and 0.2% citronella oil was applied to the tarsus of a right front leg of mosquitoes, after which the animals were placed in a bottle for 3 min. The legs were then removed, and odor preference was tested in a flight arena. (**b**) Schematic of the olfactory behavioral assay. Individual mosquitoes start each trial at the center of the odor plume, after which they are free to adjust their heading direction to navigate in or out of the plume. (**c**) An aversive index quantifying the percentage of time that mosquitoes have spent outside of the odor plume. (**d**) Mean aversive index in the last 1 s of odor application period. Mosquitoes avoid the smell of citronella oil more strongly after conditioning with a mixture of L-PDMS and citronella as compared to a mixture of glycerol and citronella oil (Wilcoxon ranksum test, p = 0.04). (**e**,**f**) Mosquitoes’ responses to linalool, a control odor not pre-exposed with oils, are similar after being conditioned with citronella oil and two different oils (Wilcoxon ranksum test, p = 0.55).

Given that L-PDMS is an aversive stimulus for mosquitoes (Fig. 1), we further tested whether it can drive aversive olfactory learning. We applied a mixture of L-PDMS and 0.2% citronella oil to the right front tarsus of mosquitoes after which the animals were rested for 3 min in a bottle (Fig. 4a). In control experiments, a mixture of glycerol and 0.2% citronella oil was applied. After letting the mosquitoes associate the smell of citronella oil and the aversive experience of L-PDMS, we released the mosquitoes from both stimuli by removing their tarsi and tested in the flight arena if the response to the citronella oil odor was altered through associative learning. Citronella oil odor was applied from a tube placed frontally to the mosquito (Fig. 4b). In each trial, an odor was applied for up to 4 s in the form of a plume extending 45 deg in azimuth. An odor was presented in closed-loop such that the mosquitoes could actively exit or re-enter the odor plume through turning (Fig. 4b). Vertical gratings were displayed and rotated according to the mosquito’s turn to provide visual feedback. The aversiveness of an odor was quantified by calculating the percentage of time that mosquitoes have spent outside of the odor plume.

We found that the aversiveness of citronella oil was higher when it was pre-exposed together with L-PDMS than when it was pre-exposed with glycerol (Fig. 4c,d). This difference was not observed for linalool, an aversive odor that was not pre-exposed (Fig. 4e,f). Similar results were observed for three other control odors that were not pre-exposed (Fig. S3). Together, these results suggest that L-PDMS acts as an aversive teaching signal for the formation of odor identity-specific memory in mosquitoes. 

## Discussion

Here we identified multifaceted functions of L-PDMS as a repellent for mosquitoes. First, we found that a mixture of L-PDMS and a topical repellent spread on a human skin had a larger effect in reducing the number of mosquito bites than the components alone. This synergistic effect was absent when a repellent was mixed with H-PDMS or glycerol, indicating that the effect is not universal for any kind of oil. Because L-PDMS but not the other two can efficiently wet mosquito, a candidate underlying mechanism is an enhanced transfer of repellents to mosquitoes. Indeed, the amount of citronellal transferred to the mosquito legs was increased almost 6-fold by the addition of L-PDMS whereas no such increase was observed with the other two oils. Similar results were obtained with DEET. This suggests that the synergistic effect can be obtained with a wider variety of repellents, as long as they are efficiently transferred to mosquito tarsi through L-PDMS and detected by sensory receptors. 

We also found that L-PDMS itself can exert lasting effects on mosquito behavior. Our previous study as well as the arm-in-cage experiment (Fig. 1) have shown that mosquitoes avoid biting a human skin treated with L-PDMS, likely to escape from a capillary force that pulls the mosquito legs towards the solution^17^. Here we found that the aversive effect of L-PDMS continues beyond the time of contact. Mosquitoes whose tarsi were treated with L-PDMS were not able to track a visual object during subsequent flight as accurately as those treated with control solutions, a disadvantage in pursuing and landing on hosts. A stronger effect was observed for ethanol that also efficiently wets mosquito legs. These results reveal a novel potential of L-PDMS and ethanol as a repellent that can compromise mosquito’s behavior for an extended period of time. The mechanism by which these solutions provide aversive effects on mosquitoes through their legs is not known. Because ethanol in particular has a property of evaporating rapidly, the experience of having their legs covered by these solutions is likely to be an aversive event for mosquitoes. It remains to be investigated if the behavioral effect of L-PDMS and ethanol extends beyond visual object tracking.

Finally, we found that the tarsal contact with L-PDMS is aversive enough for mosquitoes to form an associative olfactory memory. Mosquitoes learned to avoid the smell of citronella oil more strongly after a transient experience of having their legs covered by a mixture of citronella oil and L-PDMS. The learned avoidance was specific to citronella oil and did not generalize to other unpaired odors. This demonstrates that *Aedes albopictus* is capable of rapidly forming an olfactory memory. This ability may be utilized under natural settings as olfactory learning is observed in other mosquito species including *Culex quinquefasciatus, Anopheles gambiae,* and *Aedes aegypti*^21–25^.

## Conclusion

In sum, we show that L-PDMS can alter a sequence of mosquito behaviors and propose its practical use as a repellent acting over various time scales. By applying it on a human skin alone or together with topical repellents, L-PDMS can reduce the number of mosquito bites. When mosquitoes land on this skin, L-PDMS reduces their ability to accurately track a visual object, likely including a host, thus affecting the future identification of hosts. Furthermore, because L-PDMS confers an aversive meaning to a simultaneously presented odor, the mere presence of this conditioned odor should suffice to protect the local environment from mosquitoes following learning. In theory, the conditioned odor can be any odor including those that are safe and perceptually pleasant for humans. The results from this study will pave the way for development of innovative mosquito repellents. Adopting the skin friendly cosmetic oil L-PDMS in conjunction with low concentrations of citronella oil into mosquito repellents will protect consumers and their local environment from mosquito bites, while exhibiting hardly any odor.

## Methods

### Mosquito rearing and maintenance

All the experiments were performed on female *Aedes albopictus* 5 days after eclosion. Mosquitoes were reared at 28 °C, 70% relative humidity with a photoperiod of 12 h light and 12 h dark (lights on at 8 A.M.). Eggs were purchased from Sumika Technoservice Corporation and allowed to hatch in deoxygenated, deionized water. Larvae were fed TetraMin Baby (Spectrum Brands Holdings). Pupae were placed in a small cup of deionized water and allowed to eclose in either a 30 cm × 30 cm × 30 cm-insect cage (BugDorm-1, Mega View Science Co. Ltd) or a plastic bottle (170 ml, AS-115, Thermo Fisher Scientific Inc.). Female and male adult mosquitoes were kept in the same bottle for at least 2 days to let them mate. Mosquitoes were provided with unlimited access to 10 wt% sucrose solution (FUJIFILM Wako Pure Chemical Corporation).

### Reagents

Reagents used in all experiments were purchased from the following companies.

L-PDMS and H-PDMS (CAS No. 63148-62-9): Shin-Etsu Chemical Co. Ltd; glycerol (CAS No. 56-81-5) and ethanol (CAS No. 64-17-3): FUJIFILM Wako Chemicals Corporation; citronella oil (CAS No. 8000-29-1; 91771-61-8): Van Aroma; DEET (99.5%, CAS No.134-62-3), lactic acid (CAS No. 50-21-5), ammonia (28% water solution, CAS No. 1336-21-6), sulcatone (CAS No. 110-93-0) and linalool (CAS No. 78-70-6): Tokyo Chemical Industry Co. Ltd.

### Arm-in-cage experiments 

Two volunteers (male Asian 41Y, female Caucasian 34Y) were recruited for this study. Informed consent was received from each of the participants before the study. All mosquito landing tests using human forearms were reviewed and approved by the Ethics Committee of Kao Corporation (UMIN [K0061-2112]) in accordance with relevant guidelines and regulations in Japan and the Declaration of Helsinki.

Samples listed in Fig. 1a were applied to an area of 4 × 5 cm on the forearm of two human volunteers (male Asian 41Y, female Caucasian 34Y). The arm outside of this area was covered and thus not accessible by mosquitoes. The arm was inserted into a cage (30 × 30 × 30 cm) containing 25 female mosquitoes, which had been deprived of sugar solution for 12 h. All mosquitoes landing and initiating biting behavior during 10 min were collected and counted. For all the samples, three replicates were performed per volunteer. PDMS 6cs was selected for low viscosity PDMS and PDMS 5000cs for high viscosity PDMS.

### Analysis of repellents transferred to mosquito legs

60 mosquito legs were collected from dead mosquitoes and treated as a single sample. 2 mg/cm^2^ of solution was applied to an artificial skin surrogate (Bioskin cheek skin model, Made by Beaulax, skin type No.10C, diameter 55 mm, thickness 5 mm). The mosquito legs were individually picked up and tapped on the skin, mimicking mosquito landings. The legs were collected on a patch of glass wool with a thermal desorption tube (Gerstel K.K.) and used for GC–MS analysis. Repellents were analyzed by GC × GC-TOFMS (Pegasus 4D, Leco) coupled with a thermal desorption unit (TDU2, Gerstel K.K.). Thermal desorption was conducted at 230 °C (purge flow, 50 ml/min; purge time 3 min). Helium was used as the carrier gas at a flow rate of 0.9 ml/min in a constant flow mode. The following columns were used; the 1st column was DB-Wax (60 m × 0.25 mm i.d., 0.25 mm film thickness; J&W Scientific) and the 2nd column was DB-5 (1.29 m × 0.18 mm i.d., 0.18 mm film thickness; J&W Scientific). The temperature program for the GC oven was 40 °C for 1 min, 6 °C/min to 240 °C. Mass spectra were obtained in the electron-impact mode (70 eV). 3 replicates were obtained for each sample.

### Mosquito preparation for tethered flight

Mosquitoes were cold-anesthetized on ice, placed on a Peltier device held at 4 °C, and tethered to a stainless-steel pin (Austerlitz minutiens Φ0.1 mm) on the thorax, using an ultraviolet-curing adhesive (NOA81, Norland). Tethered mosquitoes were transferred to the virtual flight arena previously used to monitor the maneuver of *Drosophila melanogaster*^18,19^.

For the bar tracking experiment, after tethering the mosquito, 50 nl of 10% L-PDMS, 100% L-PDMS, 100% ethanol, or 100% glycerol was applied to 3–5th tarsomeres of all 6 legs.

For the olfactory behavior experiment, either a mixture of 100% L-PDMS and 0.2% citronella oil or 100% glycerol and 0.2% citronella oil was applied to the tarsus of the right front leg during cold anesthesia, after which the mosquito was placed in a bottle (170 ml, AS-115, Thermo Fisher Scientific Inc.). After 3 min of rest, the mosquito was cold anesthetized on a Peltier device, its 6 tarsi were removed, and was fixed to a pin.

### Olfactory stimulation

Odors were applied in the flight arena as described previously^18^. Briefly, air was odorized by passing it through 4 ml of odorant solution (using either mineral oil or water as a solvent) in a glass vial. The air stream was split into separate channels each lined up with a single odor vial and a solenoid valve (100E1-SR, Koganei Corporation) that regulated the open/closed state of the channel. The outputs of all channels were pooled. The stream of odors (250 ml/min) were mixed into the main air stream (1.55 l/min), and directed into the arena using ⌀6 mm Teflon tubing (main tube). A small portion of this air stream was diverted from the main tube through a ⌀2 mm outlet tube and delivered frontally to the mosquito. This configuration reduced the impact of transient changes in air pressure caused by the switching of solenoid valves and was critical not to disturb flight at the onset of odor delivery. The tip of the delivery outlet was placed 10 mm away from the mosquito. The remainder of the air stream, which was not delivered to the mosquito, was directed out of the arena and cleared using a fume hood fan unit (3-4064-11, AS ONE). Because the quantity of air reaching the mosquito decreased with increasing the suction of the fan unit, suction power was calibrated to obtain an air flow of 0.3 m/s at the mosquito location, using an air flow sensor (QB-5, Tohnic). To avoid odor contamination, Teflon was used for all parts in contact with odorized air, and tubing was periodically washed with alcohol, rinsed with purified water, and dried with clean air. The odor used were mineral oil, 100% L-PDMS, a mixture of three components (lactic acid, ammonia, sulcatone, all at 10^–6^ dilution), 0.2% citronella oil, and 1% linalool. Mineral oil was used as solvent except for a mixture of three components, which was dissolved in water.

### Visual and olfactory behavioral experiments in a virtual flight arena

Visual and olfactory behavioral experiments were conducted using the same method applied to *Drosophila melanogaster*^18^. The flight arena consisted of an odor delivery apparatus and a 24*56 array of green LEDs arranged in a half circle (Mettrix Technology Corp.^26^). LED panels were covered with 2 sheets of color filter (Roscolux #39, Rosco) to reduce the overall luminance. The display spanned ~ 210 deg horizontally and ~ 56 deg vertically below the mosquito. The entire arena was enclosed in an opaque container to prevent light entry, and the tethered mosquito was illuminated with infrared LEDs to allow visualization using a camera (Lu070M, Lumenera corporation).

Mosquitoes’ flight was monitored using two microphones (AT9904 electret condenser microphones, audio-technica) positioned laterally ~ 1 mm from the tip of the extended wing on either side of the mosquito, whose outputs were amplified (AT-MA2 amplifier, audio-technica), digitized (NI 9215, National Instruments) and analyzed in real-time using a desktop computer (Optiplex 980, Dell) to extract the turning direction and speed, which were used to update the visual and olfactory stimuli in closed-loop. The visual display and solenoid valves were controlled using a data-acquisition board and modules (NI cDAQ9178, NI 9215, NI 9264, National Instruments) and custom code written in MATLAB (MathWorks), Java, and C. Microphone signals were acquired continuously in chunks of 5 ms, and signal amplitude in each chunk was computed as the difference between the maximum and the minimum values. These amplitudes were filtered by calculating the median over the 3 most recent values and (i) summed to obtain the flight strength s, and (ii) standardized (using mean and standard deviation values computed over the previous block of trials; for the first block, running estimates computed before the experiment were used) to obtain the standard left and right wing-beat amplitudes wL and wR. Turns during the flight were assessed by monitoring the difference in wingbeat amplitudes, a proxy for yaw torque^20^. An increment Δθ in angular position was registered if the difference in standard wing-beat amplitudes Δw = wR – wL, after multiplication by the flying strength, exceeded the value of its standard deviation σΔw (computed over the previous block of trials), above which the increment was proportional to Δw, i.e., ∆θ = γ sign(∆w) max(0,|∆w|s – σ∆w), with a coupling coefficient γ = 0.375 to yield units of degrees. Angular position was computed by cumulatively summing these increments, and visual and olfactory stimuli were updated every 5 ms in accordance with the current position.

In the bar tracking experiment, two 30 deg-wide bars separated by 180 deg were set in the arena at high or low contrast. The second bar was used so that either bar was always present in the LED arena spanning ~ 210 deg. The initial position of the bars was random at the beginning of each experiment that lasted 800 s. The position of the bars was updated in closed-loop.

In the experiment involving odors, the visual stimulus was a vertical grating with a spatial frequency of 60 deg^−1^. The protocol consisted of 15 blocks, in which each of the 4 odors as well as a control odor (mineral oil) were applied in randomized order, up to a fixed duration (4 s) and in a restricted spatial region (45 deg centered at the mosquito’s heading direction at the time of odor onset). Odor application was terminated when the mosquito exited this spatial region but was re-initiated if the mosquito re-entered the region within the 4 s stimulus application period. Inter-trial interval was 10 s. When no odor was applied, air was presented in order to maintain the same flow rate. The random sequence of odor presentation was fixed across experiments and chosen not to present the same odor consecutively at the transition between successive blocks.

Experiments were initiated ~ 5 min after placing the mosquito in the flight arena, during which the position of the microphones were adjusted and the general behavior of the mosquito was observed. Mosquitoes flew in the presence of a wide-field grating controlled in closed-loop for ~ 1 min prior to the experiment. Occasionally, the air stream was turned on and off several times to trigger the flight. The experiment was terminated if the mosquito did not fly after ~ 5 min.

### Quantification and statistical analysis

All quantifications and statistical analyses were performed in Microsoft Excel and MATLAB. Statistical details for each experiment, including the statistical test performed, significance, sample size, definition of center and dispersion are reported in the corresponding figure legends. Sample sizes were set based on effect sizes and sample-by-sample variability observed in pilot experiments. In the box plot, nonoutlier range corresponds to 1.5 times the interquartile range away from the top or bottom of the box.

The bar fixation score was calculated as

$$ \left( {{\text{freq}}\left( {{\text{peak}}} \right) \, {-} \, \left( {{\text{freq}}\left( {{\text{left}}\_{\text{trough}}} \right) \, + {\text{ freq}}\left( {{\text{right}}\_{\text{trough}}} \right)} \right){/2}} \right){\text{/freq}}\left( {{\text{peak}}} \right) $$

where freq(peak) is a frequency at the peak of histogram, freq(left_trough) is a frequency at 90° left to the peak position, and freq(right_trough) is a frequency at 90° right to the peak position.

### Supplementary Information

Supplementary Figure S1.

Supplementary Figure S2.

Supplementary Figure S3.

Supplementary Information.

Supplementary Video 1.

## Data Availability

The datasets used and/or analyzed during the current study are available from the corresponding author on reasonable request.
