# Supplementary figures and images for: Hydrophobic solution functions as a multifaceted mosquito repellent by enhancing chemical transfer, altering object tracking, and forming aversive memory
Source: Sci Rep. 2024 Mar 5;14:5422. doi: 10.1038/s41598-024-55975-w (PMC10914761; doi:10.1038/s41598-024-55975-w)

figure S1. H-PDMS and glycerol do not enhance the aversiveness of ctironella oil and DEET

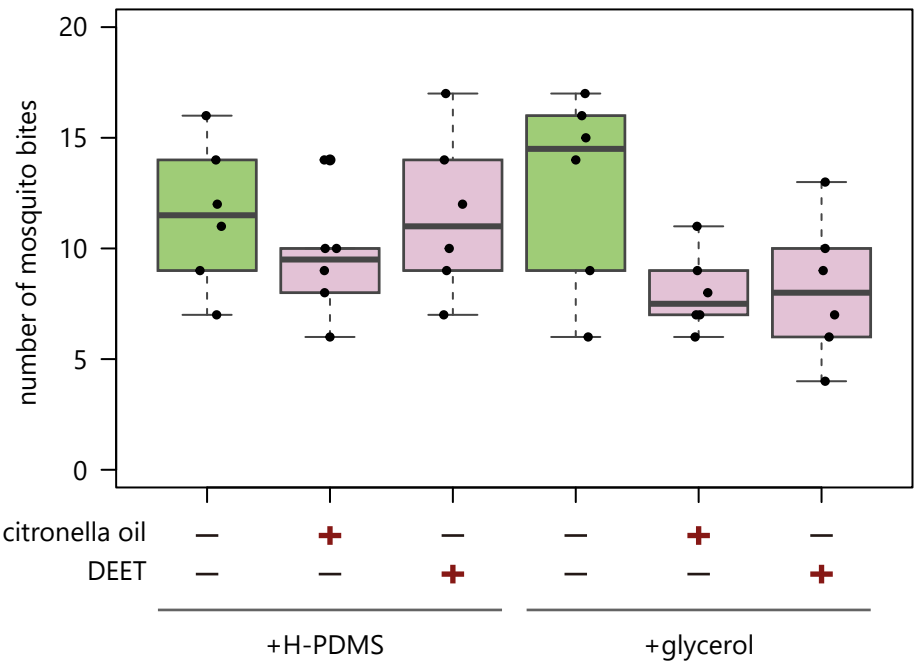

Supplement: Supplementary file 1 — Supplementary Figure S1. [file 41598_2024_55975_MOESM1_ESM.pdf]

figure 2. The effect of different oils on the direct transfer of topical repellents.

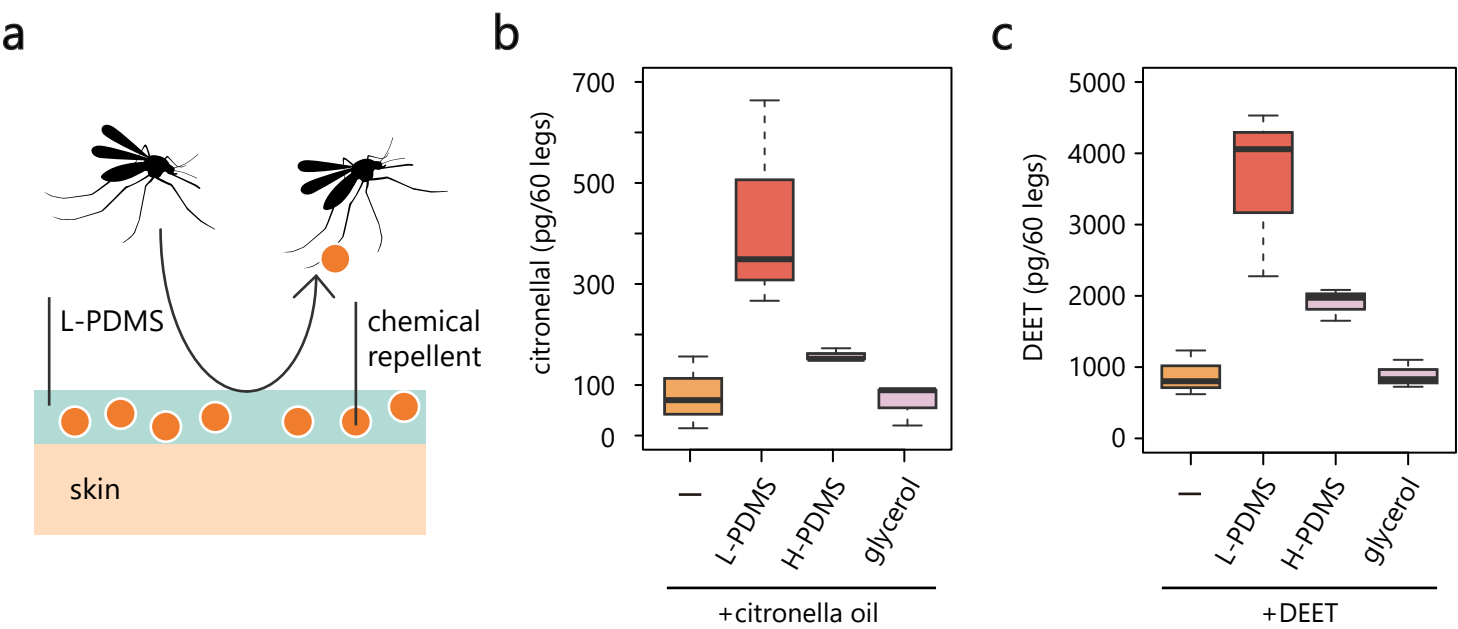

Supplement: Supplementary file 2 — Supplementary Figure S2. [file 41598_2024_55975_MOESM2_ESM.pdf]
