# Supplementary material for: Hydrophobic solution functions as a multifaceted mosquito repellent by enhancing chemical transfer, altering object tracking, and forming aversive memory
Source: Sci Rep. 2024 Mar 5;14:5422. doi: 10.1038/s41598-024-55975-w (PMC10914761; doi:10.1038/s41598-024-55975-w)

figure S3. Mosquitoes' responses to control odors are similar after being conditioned with citronella oil and two different oils

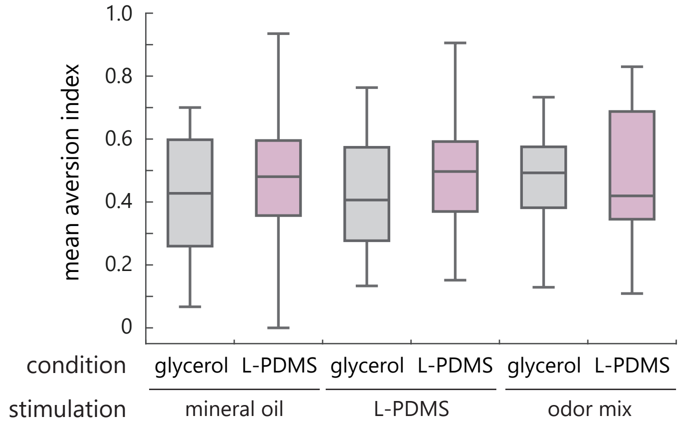

Supplement: Supplementary file 3 — Supplementary Figure S3. [file 41598_2024_55975_MOESM3_ESM.pdf]
